# Biologically Important Eremophilane Sesquiterpenes from Alaska Cedar Heartwood Essential Oil and Their Semi-Synthetic Derivatives

**DOI:** 10.3390/molecules16064775

**Published:** 2011-06-08

**Authors:** Mohammad A. Khasawneh, Yeping Xiong, Javier Peralta-Cruz, Joe J. Karchesy

**Affiliations:** 1 Department of Chemistry, College of Science, United Arab Emirates University, Al-Ain, P.O. Box 17551, UAE; 2 Department of Forest Products and Engineering, College of Forestry, Oregon State University, Corvallis, Oregon 97333, USA; Email: joe.karchesy@orst.edu (J.J.K.); 3 National School of Biological Sciences, National Polytechnic Institute, Carpio Y Plan De Ayala, Mexico City 11340 D.F. Mexico; Email: jperalta@ipn.mx (J.P.-C.)

**Keywords:** eremophilane sesquiterpenes, nootkatol, valencene-13-ol, stereochemistry, *Chamaecyparis nootkatensis*

## Abstract

The essential oil of Alaska cedar heartwood is known to contain compounds which contribute to the remarkable durability of this species. While previous research has identified several compounds, a complete description of this oil has not been undertaken. In this research a profile of the oil is given in which the major components are identified by GC, isolation and spectroscopic techniques. The major components of the steam distilled essential oil were identified as nootkatin, nootkatone, valencene, nootaktene, carvacrol, methyl carvacrol, nootkatol (**2**), and eremophil-1(10),11-dien-13-ol (**3**). The last two compounds were isolated for the first time from Alaska cedar in this research. The absolute stereochemistry at C-2 of nootkatol was shown to have the (*S*) configuration using the Mosher ester method. Assignment of stereochemistry for valencene-13-ol (**3**) was established by synthesis from valencene (**6**). Finally, two related sesquiterpenoids were synthesized from nootkatone and valencene. These sesquiterpenoids were nootkatone-1,10-11,12-diepoxide (**5**) and valencene-13-aldehyde (**4**), respectively.

## 1. Introduction

**Figure 1:**
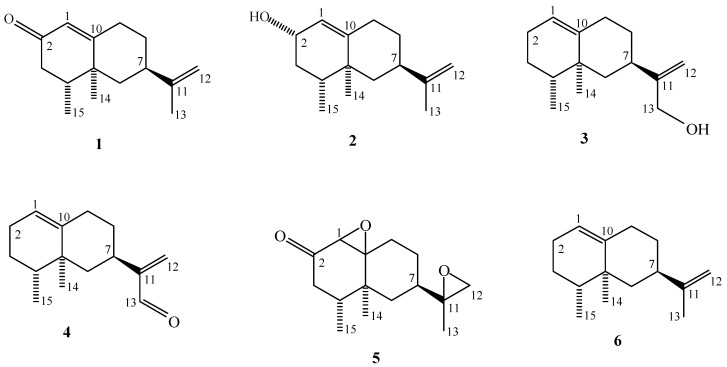
Structures of the sesquiterpenoids reported in this study.

Alaska cedar (*Chamacyparis nootkatensis*), also known as yellow cedar or Nootka cypress, is an important timber and ecological species of the costal Pacific Northwest of the United States which is known for its heartwood durability. In chemical investigations of the heartwood, carvacrol and the tropolone nootkatin were isolated and shown to be prominent components that contributed to wood durability. Subsequently the tropolone chanootin, monoterepene acids, and the eremoplilane sesquiterpenes nootkatone, nootkatene and valencene were found in early research [1]. More recently, we have isolated two monoterpenes and two sesquiterpenes from the methanol extract of Alaska cedar [2]. No GC analysis has been reported for the heartwood steam distilled essential oil. In previous research, we have reported on the bioactivities of isolated compounds and selected semi-synthetic derivatives from the heartwood essential oil of Alaska cedar against arthropods of public health importance [3,4]. In addition to the monoterpene phenol, carvacrol, the eremophilane sesquiterpenes: nootkatone (**1**), nootkatol (**2**), and valencene-13-ol (**3**) exhibited significant bioactivities (Figure 1). Semisynthetic derivatives valencene-13-aldehyde (**4**) and nootkatone-(1,10–11,12)-diepoxide (**5**) were also bioactive [3,4]. Nootkatol (**2**) and valencene-13-ol (**3**) are newly isolated from this species. While nootkatol (**2**) is a known compound, confusion in the literature required determination of stereochemistry at C-2 for the compound isolated from Alaska cedar. Valencene-13-ol (**3**) has been previously reported as an enzyme conversion product of valencene, but with no NMR data reported [5].

In this paper we wish to report on the composition of the steam distilled essential oil of Alaska cedar, isolation of compounds **2 **and **3** and their structural determination including full NMR assignments and determination of stereochemistry. We also wish to report on the preparation and NMR assignments of the semisynthetic sesquiterpenoids **4** and **5**.

## 2. Results and Discussion

### 2.1. GC Analysis of the Essential Oil of Alaska Cedar

The gas chromatogram of the crude essential oil showed several components, the major of which were identified as summarized in Table 1 by GC co-chromatography with authentic compounds or isolation and identification. The chromatogram showed that the most abundant constituents of the oil were carvacrol, nootkatene, and nootkatone. Besides nootkatin, those major constituents inevitably provoked interests of previous researchers with respect to the particular properties of Alaska cedar. Nevertheless, the GC analysis showed several unidentified compounds. The crude oil was therefore subjected to subsequent column chromatography in order to isolate the major unknown peaks shown in GC.

**Table 1:** The components of the Alaska cedar heartwood essential oil analyzed by GC.

Compounds in oil	Percentage in oil (% w/w)	Retention time (min)
4-terpineol	2.08	8.4
methylcarvacrol	1.33	9.4
carvacrol	35.37	10.8
valencene	1.50	16.4
nootkatene	20.08	17.1
nootkatol	5.20	23.5
valencene-13-ol	6.35	25.3
nootkatone	17.39	26.6
nootkatin	3.50	31.6

### 2.2. Isolation and Structure Determination of Isolated Compounds

The compositions of fractions I-VII obtained from the column chromatographic isolation of the Alaska cedar oil are given in Table 2 below.

**Table 2:** Chemical composition of fractions I through VII isolated from Alaska cedar by column chromatography.

Fractions	Main components	Weight (g)	% in total (6.2 g oil)
I	valencene, nootkatene, methyl carvacrol	1.13	18.2
II	carvacrol	2.06	33.2
III	mixture of several minor compounds	0.05	0.8
IV	**3**, nootkatone	1.80	29.1
V	mixture of several minor compounds	0.35	5.6
VI	**2**, other minor compounds	0.51	8.2
VII	**2**	0.11	1.8

In addition to previously known constituents of the oil, two compounds (**2** and **3**) were isolated from these fractions for the first time. The structural features of these two compounds are discussed in the following subsections.

#### 2.2.1. Nootkatol (eremophil-1(10),11-dien-2-ol) (**2**)

This compound was isolated as a white amorphous powder, with [*α*]^25^_D_ + 203° (*c* 0.5, CHCl_3_), IR (3440cm^−1^, 1620cm^−1^, 1510cm^−1^) and melting point was 65 °C (uncorrected). HRCIMS indicated that the molecular formula was C_15_H_24_O since it gave *m/z* 220.18165 and C_15_H_24_O requires 220.18272. ^1^H- and ^13^C-NMR assignments are summarized in Table 3. The ^13^C-NMR showed signals of 15 carbons at 150.6, 146.5, 124.7, 108.9, 68.4, 45.0, 41.2, 39.7, 38.6, 37.6, 33.3, 32.8, 21.2, 18.6, and 15.8 ppm, indicating four signals in the resonance region of double bonds and one signal for the carbon connected to a hydroxyl group.

DEPT 90° and 135° experiments confirmed the presence of three quaternary carbons (150.6, 146.5, 38.6 ppm), four methine carbons (124.7, 68.4, 41.2, 39.7 ppm), five methene carbons (108.9, 45.0, 37.6, 33.3, and 32.8 ppm), and three methyl carbons (21.2, 18.6 and 15.8 ppm). ^1^H- and ^13^C-NMR data are in good agreement with the literature values reported for nootkatol [5,6,7]. 2-D NMR data added further confirmation of the assignments shown in Table 3.

**Table 3:** ^1^H- and ^13^C-NMR (in CDCl_3_, Bruker Model AM 400 MHz for ^1^H and 100 MHz for ^13^C) assignments for compound **2** (nootkatol). s = singlet, d = doublet, t = triplet, m = multiplet.

Carbon	^13^C	^1^H
1	124.7	5.32, 1H, d, *J* = 1.6 Hz
2	68.4	4.25, 1H, m
3	37.6	(a) 1.76, 1H, td, *J* = 2.0, 6.5 Hz
		(b) 1.37, 1H, td, *J* = 12.4, 10.0 Hz
4	39.7	1.51, 1H, br d, *J* = 2.1 Hz
5	38.6	—
6	45.0	(a) 1.85, 1H, dd, *J* = 12.6, 2.7 Hz
		(b) 0.95, 1H, m, *J* = 2.7 Hz
7	41.2	2.25, 1H, tt, *J* = 12.4, 3.0 Hz
8	32.8	(a) 2.33, 1H, m
		(b) 2.1, 1H, ddd, *J* = 14.1,4.2, 2.6 Hz
9	33.3	(a) 1.79, 1H, dd, *J* = 2.0, 4.5 Hz
		(b) 1.20, 1H, dm, *J* = 4.3 Hz
10	146.5	—
11	150.6	—
12	108.9	4.68, 2H, br s
13	21.2	1.71, 3H, s
14	18.6	0.99, 3H, s
15	15.8	0.89, 3H, d, *J* = 6.9 Hz

Both C-2 epimers of nootkatol were reported in the literature by the reduction of nootkatone with lithium aluminum hydride [6]. Another report on the reduction of nootkatone to produce both epimers of nootkatol was made by Ohizumi *et al.* [7]. Later on, nootkatol was produced along with compound **3** and nootkatone by the enzymatic hydroxylation of valencene using chicory (*Cicorium intybus* L.) roots [5].

By reviewing the above references, one can conclude that there is some confusion in the literature about the absolute configuration of the alcohol moiety at C-2 of the natural product, which was sometimes called nootkatol [5,8] and sometimes epinootkatol [7,9,10]. To clarify this point in the literature, a clear-cut method with the ability to unambiguously determine the absolute configuration of C-2 on nootkatol needs to be implemented. The modified Mosher method described by Ohtani *et al.* [11] was applied for this purpose. In this method, both the (*S*)- and (*R*)-2-methoxy-2-(trifluromethyl)-2-phenylacetic acid (MTPA) esters of nootkatol isolated from Alaska cedar were synthesized and their ^1^H-NMR spectra were recorded.

According to a model proposed by Mosher *et al.* in which the methine proton, ester carbonyl and trifluromethyl groups of the MTPA moiety lie in the same plane in solution [12,13] and for convenience this plane is called the MTPA plane. Since it is possible to correctly predict the MTPA plane of the ester and due to the anisotropic effect of the phenyl ring, the upfield shift of protons on both sides of the MTPA plane will depend on the configurations of the alcohol as well as the MTPA moiety. The parameter Δδ, which is defined arbitrarily as δ_S_-δ_R_, can be used as a guide to determine the absolute configuration of the hydroxyl group as follows. Protons with positive Δδ values should be placed on the right side of the MTPA plane and those with negative Δδ values to the left using the Mosher model.

**Figure 2:**
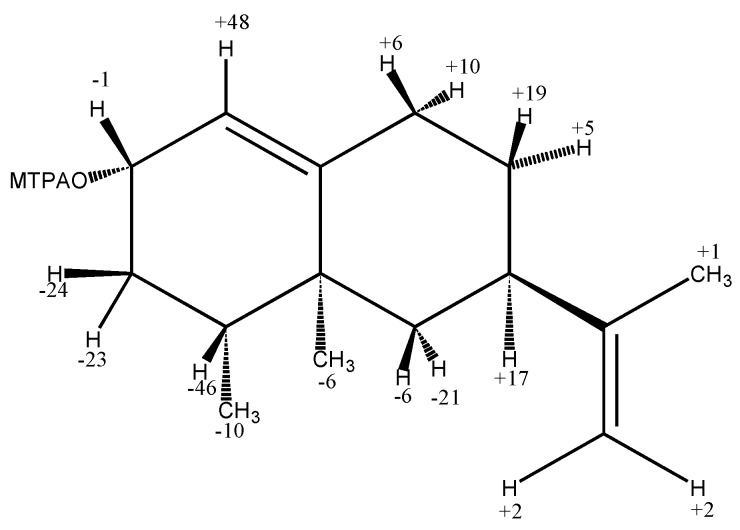
Δδ values (in Hz) for nootkatol (**2**) isolated from Alaska cedar (Δδ = δ_S_ − δ_R_).

The values for Δδ (in units of Hz) were calculated for each of the protons in nootkatol (Figure 2, Δδ values on the structure). If nootkatol is portrayed according to the Mosher model, then protons H-3, H-6, H-14 and H-15 lie to the left of the MTPA plane (Δδ values are negative) and H-1, H-7, H-8, H-9 (and to a much lesser extent H-12 and H-13) lie to the right of the MTPA plane (Δδ values are positive). From these results, it can be concluded that the alcohol in nootkatol has an α-orientation and therefore the absolute configuration at C-2 is (*S*) as shown in Figure 1.

#### 2.2.2. Eremophil-1(10),11-dien-13-ol (**3**)

EIMS of this compound exhibited a strong M^+^ molecular ion peak at *m/z* 220 indicating that the molecular weight was 220. High resolution MS indicated that the molecular formula was C_15_H_24_O since it gave *m/z* 220.18271 and C_15_H_24_O requires 220.18272. ^1^H- and ^13^C-NMR assignments are summarized in Table 4. The ^13^C-NMR shows signals of 15 carbons at 154.5, 143.2, 120.8, 108.3, 65.7, 45.8, 41.3, 38.3, 37.1, 34.0, 33.1, 27.5, 26.3, 18.8, and 16.0 ppm, indicating four signals in the resonance region of double bonds and one signal for the carbon connected to a hydroxyl group. Comparing to the ^13^C-NMR of valencene and other related eremophilane compounds those data (shown in Table 4) displayed very similar patterns, indicating that the unknown compound has the same skeleton as valencene and belong to the eremophilane group. DEPT 90° and 135° experiments confirmed the presence of three quaternary carbons (154.5, 143.2, 38.3 ppm), three methine carbons (120.8, 41.3, 37.1 ppm), seven methene carbons (108.3, 65.7, 45.8, 34.0, 33.1, 27.5, and 26.3 ppm), and two methyl carbons (18.8 and 16.0 ppm). Those data obviously confirmed the assignments: C-1, 120.8; C-12, 108.3; C-13, 65.7; C-5, 38.3; C-10 or C-11, 154.5 or 143.2; C-14 or C-15, 18.8 or 16.0 ppm. The ^1^H spectrum showed such characteristic chemical shifts for the protons in the double bonds as H-1 at 5.33(t), H-12 at 5.02(s) and 4.88(s), and H-13 at 4.12(s), and that was also confirmed by HSQC (Heteronculear Single Quantum Coherence), in which ^1^H-^13^C one bond correlation is observed. Furthermore, one singlet at 0.95 ppm and one doublet (*J* = 6 Hz) at 0.87 ppm shown in the ^1^H spectrum also suggests the presence of a secondary methyl group (C-15) and a tertiary methyl group (C-14). 

**Table 4:** ^1^H and ^13^C-NMR (in CDCl_3_, Bruker Model AM 400 MHz for ^1^H and 100 MHz for ^13^C) assignments for **3** (eremophil-1(10),11-dien-13-ol). s = singlet, d = doublet, t = triplet, m = multiplet.

carbon	^13^C (ppm)	^1^H (ppm)
1	120.8	5.33, 1H, t, *J* = 2.4 Hz
2	26.3	2.01, 2H, m
3	27.5	1.41-1.47, 3H, m
4	41.3	
5	38.3	—
6	45.8	(a) 1.01, 1H, d, *J* = 12.6 Hz
		(b) 1.93, 1H, m
7	37.1	2.31, 1H, m
8	33.1	(a) 2.09, 1H, td, *J* = 13.4, 3.4 Hz
		(b) 2.31, 1H, m
9	34.0	(a) 1.82, 1H, m
		(b) 1.21, 1H, dd, *J* = 4.1, 13.3 Hz
10	143.2	—
11	154.5	—
12	108.3	(a) 5.02, 1H, s
		(b) 4.88, 1H, s
13	65.7	4.12, 2H, s
14	18.8	0.95, 3H, s
15	16.0	0.87, 3H, d, *J* = 6.0 Hz

HMBC (Heteronuclear Multiple Bond Correlation) shows ^1^H-^13^C long-range correlations. The correlations of H-13/C-13 and H-14/C-10 were observed from the HMBC spectrum, indicating that C-11 had a resonance signal at 154.5 ppm and the methyl group at C-14 had a single proton signal at 0.95 ppm. Therefore, the two methyl groups at C-14 and C-15 had the ^13^C signal at 18.8 and 16.0 ppm, respectively, according to HSQC spectrum. HMBC spectrum also showed the correlation of methyl hydrogens at C-14 to C-5, and methyl hydrogens at C-15 to C-4.

The proton signal at 4.12 ppm (H-13) had a correlation with three carbon signals at 37.1, 108.3, 154.5 ppm from HMBC spectrum, indicating that C-7 had an assignment at 37.1 ppm since C-11 and C-12 had chemical shifts at 154.5 and 108.3 ppm, respectively. The proton signal at 0.87 ppm (H-15) had a correlation with three carbon signals at 27.5, 38.3, 41.3 ppm from HMBC spectrum, indicating that C-3 had an assignment at 27.5 ppm since C-4 shifted at 41.3 ppm and C-5 at 38.3 ppm. Long range connection between the proton at 0.95 ppm (H-14) and C-4, C-5, C6, C-10 also can be seen from HMBC spectrum, and thus the C-6 signal was assigned to the chemical shift at 45.8 ppm.

The remaining assignments for carbons (C-2, C-8 and C-9) and protons given in Table 4 are based on all NMR spectra, and especially, those assignments all agree with the COSY spectrum, which shows the coupling relationships between correlated protons.

Stereochemistry shown by difference Nuclear Overhauser Effect (NOE) indicates H-7 and the methyl group at C-14 is on the same side of ring. An enhancement (3.15%) at H-14 (0.95 ppm) is observed upon irradiation of H-7 at 2.31 ppm and hence the isopropen-2-ol group is at equatorial position. Further evidence for this conclusion was achieved by semisynthesis of **3** from the well established valencene (**6**) in two steps. The semisynthetic product exhibited identical MS, NMR and optical rotation data as those of the natural product, which proved that the original assignment of C-7 was correct. For example, in the ^13^C-NMR spectrum of three carbons 11 and 13 exhibited an expected increase in their chemical shifts to 154.5 and 65.7 as opposed to 150.2 and 20.8, respectively for the same carbons in **6**. Therefore, the assignment of stereochemistry around C-7 is (*R*) and the name of this compound is (4*R*,5*S*,7*R*)-valencene-13-ol or (4*R*,5*S*,7*R*)- eremophil-1(10)-,11-dien-13-ol.

### 2.3. Synthesis

#### 2.3.1. Synthesis of nootkatone-1,10-11,12-diepoxide (**5**)

This compound was synthesized from nootkatone-11,12-epoxide obtained from nootkatone as outlined in the Experimental section. Its molecular formula was deduced to be C_15_H_22_O_3_ based on HRCIMS. The corresponding ^1^H-NMR spectrum revealed the absence of any vinylic protons in this molecule. The three methyl groups are present at their expected position for this compound δ 1.28 (3H, s, H-13), 0.93 (3H, s, H-14), 0.78 (3H, d, *J* = 6.8 Hz, H-15). Similar to nootkatone 1,10-epoxide, H-1 appeared at δ 3.08 (1H, s) in the diepoxide. The ^13^C-NMR spectrum confirmed the disappearance of all alkenic carbons and the emergence of four sp^3^ hybridized carbons attached to oxygen at δ 63.0 (C-1) 69.2 (C-10), 54.3 (C-11), 60.0 (C-12). The ketonic carbon at δ 207.3 (C-2) was, as expected, in complete agreement with that of nootkatone-1,10-epoxide. The ^13^C-NMR spectrum of nootkatone diepoxide in fact showed two sets of signals for many of the carbon atoms. This can be explained by the expected synthesis of two diastereomeric forms of **5**.

#### 2.3.2. Synthesis of valencene-13-aldehyde (**4**)

This compound was synthesized from valencene as outlined in the Experimental section. The molecular formula of this compound was established based on HRCIMS to be C_15_H_22_O. Its ^1^H-NMR spectrum indicated the emergence of aldehyde proton at δ 9.45 (1H, s, H-13), three vinylic protons at 6.20 (1H, s, H-12 cis to CHO), 5.85 (1H, s, H-12 trans to CHO), 5.25 (1H, brs, H-1). The last three signals appeared at reasonably close chemical shifts as those calculated using NMR tables, respectively at δ 6.09, 5.87 and 5.12. The ^13^C-NMR spectrum showed the emergence of an aldehyde carbon at δ 195.0 (C-13) in addition to the four alkenic carbons at δ 120.9 (C-1), 142.9 (C-10), 155.6 (C-11), 133.2 (C-12). Compared to valencene starting material C-1 and C-10 occurred at virtually the same chemical shift (120.9 and 142.9 in valencene), but C-11 and C-12 have been shifted dramatically from δ 150.2 and 108.5 in valencene to 155.6 and 133.2 in **4**, respectively. These considerations provide a compelling evidence for the structure of this compound as valencene-13-aldehyde.

## 3. Experimental

### 3.1. General

Hexane (EM Science, Gibbstown, NJ 99.4%) was used as received. Acetone (99.6%), ethyl acetate (99.9%), dichloromethane (99.9%) and methanol (99.9%) were all purchased from Fisher Scientific, Fair Loan, NJ and distilled over anhydrous Na_2_SO_4_ prior to use. Diethyl ether (EM Science, Gibbstown, NJ 98%) was used as received. Deuterated chloroform (Aldrich Chemical Company, Milwaukee, WI, 99.9 atom %D) was used as received for NMR experiments. SeO_2_ (Matheson Company, Inc., East Rutherford, NJ), NaBH_4_ (Mallinckrodt, Paris, KY 96%, H_2_O_2_ (Mallinckrodt, 30% solution in water), triethylamine (EM Science, 98% min), pyridine (Fisher, 99.9%), Na_2_CO_3_ (Mallinckrodt, 99.7%), NaHCO_3_ (Mallinckrodt, 99.7%), (R)-(−)-α-methoxy-α-trifluoromethyl acetic acid chloride (Fluka, °99%), (S)-(+)-α-methoxy-α-trifluoromethyl acetic acid chloride (Fluka, °99%) were all used as received from vendors. (+)-Valencene (Fluka Chemical Company, Switzerland) and (+)-nootkatone (Lancaster, Pelham, NH) were purified by silica gel columns prior to use and NMR spectroscopy was used to check purity. Anhydrous Na_2_SO_4_ (Fisher Scientific, Fair Lawn, NJ, 100%), silica gel (EM Science, particle size 0.063–0.200 mm, 70–230 mesh ASTM), TLC plates (EM Science, Kieselgel 60 F_254_) were all used as received. All NMR measurements were recorded using a Bruker Avance NMR (400 MHz in the case of ^1^H and 100 MHz in the case of ^13^C) spectroscopy instrument.

### 3.2. Plant Material and Essential Oil

Alaska cedar was obtained from the Hungry Mountain area in the Sol Duc River drainage of the Olympia National Forest, Washington State (Oregon State University Herbarium voucher specimen #188046). The ground heartwood (1.5 kg) was steam distilled in a glass distillation apparatus for 12 h to give 26 g of essential oil (1.73% yield).

### 3.3. Analysis of Essential Oil

A gas chromatograph (GC-17A Shimadzu, Japan) was used for monitoring composition of fractions and identifying pure compounds by using standards. The gas chromatograph was equipped with flame ionization detector (FID). The column (30 m × 0.25 mm DB-5, 0.25 μm, J&W Scientific) was temperature programmed from 100 °C for 1 min, then to 150 °C at a rate of 5 °C/min, then to 220 °C at 3 °C/min, and finally to 240 °C at 5 °C/min and held at that temperature for 2 min.

### 3.4. Chromatographic Fractionation of the Essential Oil

The distilled oil (6.2 g) was dissolved in hexane (5.0 mL) and chromatographed over a silica gel column using a gradient solvent mixture of hexane and diethyl ether from 100% hexane to 60:40 (hexane/diethyl ether, v/v). Eluent aliquots of 20 mL were collected with a Gilson FC-100 fraction collector and monitored by GC and TLC developed with dichloromethane, and the plates were visualized under UV light and subsequently sprayed with acidic vanillin solution followed by heating. Aliquots of eluent with same component checked by TLC were combined together to form one fraction. Seven major fractions were obtained with their chemical compositions as shown in Table 2 using GC and TLC against standards of the known compounds. **2** and **3** were isolated by repeated silica gel columns of fractions IV and VII.

### 3.5. Synthesis of Nootkatol-(R)-α-methoxy-α-(trifluoromethyl)phenyl acetate and Nootkatol-(S)-α-methoxy-α-(trifluoromethyl)phenyl acetate

To a stirred solution of nootkatol (6 mg, 0.028 mmol) in CH_3_Cl (0.5 mL) at room temperature was added triethylamine (5.5 mg, 0.054 mmol) followed by (*S*)-α-methoxy-α-(trifluoromethyl)phenyl-acetyl chloride (6.8 mg, 0.027 mmol) as a solution in CH_3_Cl (1.0 mL) and the resultant mixture was stirred for 24 h. The reaction mixture was washed with ice cold water (3.0 mL), 2.0 M HCl (1.0 mL), saturated NaHCO_3_ solution (3.0 mL) and the organic layer was dried (anhydrous Na_2_SO_4_), and concentrated in vacuum to yield the title product (12.2 mg, 0.028 mmol, 100% yield). The product was purified by using a mini silica column with hex-EtOAc (90:10) as eluent. The same procedure was followed to synthesize the (*S*) enantiomer using (*R*)-α-methoxy-α-(trifluoromethyl)phenylacetyl chloride as a reagent.

### 3.6. Synthesis of Valencene-13-aldehyde (4)

To a stirred solution of valencene (1.00 g, 4.89 mmol, 1.00 equiv.) dissolved in freshly distilled pyridine (10 mL) at room temperature was added SeO_2_ (1.00 g, 9.01 mmol, 1.84 equiv.). The resultant yellow mixture was refluxed for 5 h until it turned black. The mixture was then filtered to remove the selenium dust and passed through a funnel packed with a mixture of 1:1 silica gel:Na_2_CO_3_ (w/w) washing with diethyl ether. The filtrate was concentrated in vacuum to remove the diethyl ether and pyridine was removed by vacuum distillation. The resulting oil was chromatographed (silica gel-Na_2_CO_3_ 1:1 mixture, elution with hexane to give the title compound (0.125 g, 0.572 mmol, 12% yield).

Data for **4**: yellow oil; (R_f_ = 0.2, hexane 100%); HRCIMS [M]^+^ 218.16711 calculated for C_15_H_22_O, requires 218.16706. Selected ^1^H-NMR spectral data (CDCl_3_, 400 MHz) δ 9.45 (1H, s, H-13), 6.20 (1H, s, H-12 cis to CHO), 5.85 (1H, s, H-12 trans to CHO), 5.25 (1H, brs, H-1). ^13^C-NMR data (CDCl_3_, 100 MHz) δ 120.9 (C-1), 26.3 (C-2), 27.5 (C-3), 41.3 (C-4), 38.4 (C-5), 45.3 (C-6), 31.8 (C-7), 32.9 (C-8), 33.5 (C-9), 142.9 (C-10), 155.6 (C-11), 133.2 (C-12), 195.0 (C-13), 16.0 (C-14), 18.6 (C-15).

### 3.7. Synthesis of Nootkatone-diepoxide (5)

To a cold solution of nootkatone-11,12-epoxide (1.70 g, 7.26 mmol, 1.00 equiv.) in anhydrous methanol (50 mL) was added H_2_O_2_ (1.48 g, 30% by weight, 43.56 mmol, 4.94 mL, 6.00 equiv.) followed by 6.0N KOH (2.5 mL) over a period of 10 min and the resultant mixture was stirred for 3 h. The reaction mixture was then quenched with cold deionized water (30 mL), and methanol was eliminated in vacuum. The product was extracted from the aqueous solution by diethyl ether (3 × 20 mL). The combined ethereal layer was dried (Na_2_SO_4_), filtered, and concentrated under vacuum to yield the title compound (0.735 g, 40.5% yield). NMR spectrum showed a mixture of diastereomers with high purity.

Data for **5**: Semisolid residue; (R_f_ = 0.50, hex:acetone 50:50 v/v); HRCIMS [M]^+^ 250.15670 calculated for C_15_H_22_O_3_, requires 250.15689. Selected ^1^H-NMR data (CDCl_3_, 400 MHz) δ 3.08 (1H, s, H-1), 1.28 (3H, s, H-13), 0.93 (3H, s, H-14), 0.78 (3H, d, *J* = 6.8 Hz, H-15). ^13^C-NMR data (CDCl_3_, 100MHz) δ 63.0 (C-1), 207.3 (C-2), 42.3 (C-3), 39.9 (C-4), 36.8 (C-5), 38.0 (C-6), 32.8 (C-7), 30.1 (C-8), 26.7 (C-9), 69.2 (C-10), 54.3 (C-11), 60.0 (C-12), 18.3 (C-13), 15.3 (C-14), 14.8 (C-15).

## 4. Conclusions

An analysis of the steam distilled essential oil from the heartwood of Alaska cedar was undertaken in this paper. The oil was shown to contain, among others, nine major compounds: 4-Terpineol, methyl carvacrol, carvacrol, nootkatin, valencene, nootkatene, nootkatone, nootkatol (**2**) and valencene-13-ol (**3**) as major components of this essential oil.

The absolute stereochemistry at C-2 of nootkatol was shown to have the (*S*) configuration using the Mosher ester method. Assignment of stereochemistry for valencene-13-ol (**3**) was established by synthesis from valencene (**6**). Additionally, two related sesquiterpenoids were synthesized from nootkatone and valencene. These sesquiterpenoids were respectively nootkatone-1,10-11,12-diepoxide (**5**) and valencene-13-aldehyde (**4**).
